# Hydrogen Sulfide Protects Retinal Pigment Epithelial Cells from Oxidative Stress-Induced Apoptosis and Affects Autophagy

**DOI:** 10.1155/2020/8868564

**Published:** 2020-12-30

**Authors:** Liming Hu, Jia Guo, Li Zhou, Sen Zhu, Chunming Wang, Jiawei Liu, Shanshan Hu, Mulin Yang, Changjun Lin

**Affiliations:** School of Life Sciences, Lanzhou University, Lanzhou, China

## Abstract

Age-related macular degeneration (AMD) is a major cause of visual impairment and blindness among the elderly. AMD is characterized by retinal pigment epithelial (RPE) cell dysfunction. However, the pathogenesis of AMD is still unclear, and there is currently no effective treatment. Accumulated evidence indicates that oxidative stress and autophagy play a crucial role in the development of AMD. H_2_S is an antioxidant that can directly remove intracellular superoxide anions and hydrogen peroxide. The purpose of this study is to investigate the antioxidative effect of H_2_S in RPE cells and its role in autophagy. The results show that exogenous H_2_S (NaHS) pretreatment effectively reduces H_2_O_2_-induced oxidative stress, oxidative damage, apoptosis, and inflammation in ARPE-19 cells. NaHS pretreatment also decreased autophagy levels raised by H_2_O_2_, increased cell viability, and ameliorated cell morphological damage. Interestingly, the suppression of autophagy by its inhibitor 3-MA showed an increase of cell viability, amelioration of morphology, and a decrease of apoptosis. In summary, oxidative stress causes ARPE-19 cell injury by inducing cell autophagy. However exogenous H_2_S is shown to attenuate ARPE-19 cell injury, decrease apoptosis, and reduce the occurrence of autophagy-mediated by oxidative stress. These findings suggest that autophagy might play a crucial role in the development of AMD, and exogenous H_2_S has a potential value in the treatment of AMD.

## 1. Introduction

Age-related macular degeneration (AMD) is the leading cause of irreversible vision loss in the elderly people around the world, and the prevalence of AMD is increasing [1]. Although the formation mechanism of AMD remains to be revealed, it is clear that oxidative damage of retinal pigment epithelial (RPE) cells contributes significantly to AMD [2]. The retina is one of the most oxygen-consuming tissues in the human body, and RPE cells are particularly vulnerable to oxidative stress caused by reactive oxygen species (ROS). Intracellular enzymes, such as catalase, superoxide dismutase (SOD), and glutathione peroxidase (GPx), protect RPE cells against oxidative stress through scavenging ROS and attenuating oxidative damage. Further research reveals that several antioxidants could inhibit AMD progression [1–3]. Therefore, inhibiting oxidative stress-induced RPE cell damage might represent an effective approach to slow down the progress of AMD in patients [1, 3].

Autophagy, a proteolytic system, plays an important role in maintaining RPE cell functions and homeostasis since these cells are exposed to sustained oxidative stress. Many studies report that autophagy occurs in RPE cells [4, 5] and is associated with the pathogenesis of many human diseases, including cancer, diabetes, neurodegenerative disorders, and AMD. The impairment of autophagy in RPE cells could lead to the accumulation of damaged organelles and various toxic proteins, including lipofuscin, and promote the formation of drusen, a typical phenomenon of AMD [5, 6]. Some studies reveal that autophagy significantly increases after RPE cells were exposed to oxidative stress [4]. Nevertheless, it remains unclear whether oxidative stress-triggered autophagy has the effect of slowing down or speeding up the progress of AMD.

Hydrogen sulfide (H_2_S) is an important intracellular gaseous mediator, analogous to nitric oxide and carbon monoxide, which was synthesized in cells by multiple enzymes. In recent years, H_2_S has been recognized to play an essential role in the pathophysiological process of various tissues and organs in mammals, especially against oxidative stress [7–11]. H_2_S could scavenge intracellular superoxide anions and hydrogen peroxide directly [12]. H_2_S has been reported to have diverse physiologic functions, such as vasodilatation, lowering blood pressure, anti-inflammation, anti-cancer, and reducing oxidative stress [11, 13]. Moreover, H_2_S is produced in retinal tissue and attenuates high glucose-induced human retinal pigment epithelial cell inflammation by inhibiting ROS formation [12], but some studies also illustrate H_2_S-caused retinopathy [14, 15].

In this study, we investigate how oxidative stress impacts ARPE-19 cells by altering autophagic flux and whether exogenous H_2_S protects ARPE-19 cells against H_2_O_2_-induced oxidative damage.

## 2. Materials and Methods

### 2.1. Materials

ARPE-19 cell lines were purchased from China Center of Type Culture Collection (Shanghai, China). DMEM medium was obtained from Hyclone (Beijing, China). Fetal bovine serum was purchased from Tianhang Biotechnology (Hangzhou, China). Hydrogen peroxide was purchased from Damao Chemical Reagent Factory (Tianjin, China). Sodium hydrosulfide was obtained from Macklin (Shanghai, China). Anti-LC3B antibody and anti-P62 antibody were purchased from Cell Signaling Technology (Danvers, MA, USA). Anti-GAPDH antibody was purchased from Santa Cruz Biotechnology (CV, USA). Polyoxymethylene was obtained from Spectrum Chemical MFG. Corp. (Shanghai, China). Annexin V-FITC/PI apoptosis detection kit, caspase 3 activity assay kit, and Ad-mCherry-GFP-LC3B were purchased from Beyotime (Shanghai, China). Cell malondialdehyde assay kit, superoxide dismutase assay kit, and reduced glutathione assay kit were purchased from Nanjing Jiancheng Bioengineering Institute (Nanjing, China). TNF-*α* ELISA kit and IL-1*β* ELISA kit were obtained from Bioswamp (Wuhan, China). Hoechst 33342 and PI were purchased from Sangon Biotech (Shanghai, China). 3-(4,5-Dimethylthiazol-3-yl)-2,5-diphenyl tetrazolium bromide was purchased from Solarbio Life Science (Beijing, China). The autophagy inhibitor 3-MA was purchased from Santa Cruz Biotechnology (CV, USA). Baf A1 (inhibiting the fusion of autophagic vesicles and lysosomes) was purchased from Sangon Biotech (Shanghai, China). 2′,7′-Dichlorofluorescin diacetate was purchased from Sigma-Aldrich (St. Louis, MO, USA). Other common reagents used in the study are of analytical purity grade.

### 2.2. Cell Culture and Treatment

Human ARPE-19 cells were cultured in DMEM high-glucose medium supplemented with 10% FBS, 100 U/mL penicillin, and 100 mg/mL streptomycin at 37°C in air containing 5% CO_2_. To induce cellular oxidative stress, cells were treated with hydrogen peroxide, when cells reached 70%-80% confluence. Different drug concentrations were used to test the toxicity of H_2_O_2_ (0-1600 *μΜ*) and NaHS (0-3200 *μΜ*). Cells were pretreated with NaHS (800 *μΜ*) for 30 min and then coincubated with H_2_O_2_ for 24 h to evaluate the effects of H_2_S. To detect the role of autophagy, cells were pretreated with the autophagy inhibitor 3-MA for 3 h and then coincubated with H_2_O_2_ for 24 h. ARPE-19 cells are adherent cells, and cells need to be trypsinized before being collected by centrifugation, if no special instructions.

### 2.3. Measurement of ROS Production

The ARPE-19 cells (1 × 10^5^ cells/well) were seeded in 6-well plates for 24 h. After that, cells were treated with DFCH-DA for 30 min in the dark. Then, cells were pretreated with or without NaHS for 30 min and subsequently coincubated with or without H_2_O_2_ for 1 h. Then, the treated cells were collected to detect intracellular ROS by flow cytometry. Untreated cells were used as the control, and cells were treated with H_2_O_2_ for 1 h as a positive control. Data were collected from at least 10,000 cells. The results were analyzed by FlowJo software.

### 2.4. Apoptosis Rate Detection with Annexin V-FITC/PI by Flow Cytometry

The ARPE-19 cells (1 × 10^5^ cells/well) were seeded in 6-well plates for 24 h. After that, cells were pretreated with NaHS for 30 min, and subsequently treated with H_2_O_2_ for another 24 h. Then, cells were collected and rinsed three times with PBS before stained with Annexin V-FITC/PI staining. Four hundred *μ*L binding buffer, 5 *μ*L Annexin V-FITC, and 10 *μ*L PI were added to each sample, respectively. Cells were incubated at room temperature for 10 min in the dark before flow cytometric assay. Data were collected from at least 10,000 cells, and the percentage of apoptosis cells in each sample was recorded by flow cytometry and analyzed by FlowJo software.

### 2.5. Western Blot Analysis

The ARPE-19 cells were pretreated with or without NaHS or 3-MA for the suggested time and subsequently treated with H_2_O_2_ for 1 h or the suggested time. In another experiment, cells were pretreated with Baf A1 for 1 h and subsequently treated with NaHS for 24 h. After that, cells were collected and lysed in RIPA buffer containing 1% protease inhibitor PMSF for 30 min on ice. After centrifugation at 13201 g for 10 min at 4°C, the supernatant was collected. And then, equal amounts of protein lysates were loaded in each lane, separated on 12% SDS-PAGE gel, and subsequently transferred to a PVDF membrane. The membrane was blocked with 5% skimmed milk powder solution for 1 h at room temperature and incubated with primary antibodies overnight at 4°C. After being washed by PBS with 0.1% Tween-20, the membrane was treated with horseradish peroxidase-conjugated second antibodies for 1 h at room temperature. The protein concentration was quantified by using the BCA protein assay kit. GAPDH was used as the internal control to confirm equal protein loading. Protein bands were visualized and analyzed by a chemiluminescence system.

### 2.6. MTT Assay of Cell Viability

The 3-(4,5-dimethylthiazol-3-yl)-2,5-diphenyl tetrazolium bromide (MTT) assay was used to test the effects of H_2_O_2_ and NaHS on cell viability. In brief, APRE-19 cells were cultured in 96-well plates (1 × 10^5^ cells/well) and then treated with H_2_O_2_ (0-1600 *μ*M) or NaHS (0-3200 *μ*M) for 24 h. To detect the effect of NaHS, cells were pretreated with NaHS for 30 min and then coincubated with H_2_O_2_ for 24 h. Then, cell medium was replaced with equal complete medium containing 1 mg/mL MTT and cells were incubated at 37°C for another 4 h. Then, the medium was poured off, and DMSO was added to dissolve crystal violet. The absorbance was detected at 470 nm by the microplate reader.

### 2.7. Transmission Electron Microscopy (TEM)

ARPE-19 cells were seeded in 6-well plates, pretreated with NaHS for 30 min, and then incubated with H_2_O_2_ for 24 h. Cells were collected by centrifugation after drug treatment and fixed with 2.5% special glutaraldehyde at 4°C overnight. After being washed with PBS 3 times, cells were fixed with 1% osmium tetroxide at 4°C for another 4 h. Then, cells were dehydrated in gradient concentrations of ethanol and switched to acetone and subsequently embedded in epoxy resin (SPI-PON-812) and polymerized in epoxy resin at 70°C overnight. Then, the samples were sliced into ultrathin sections (50 nm), stained with uranyl acetate and lead citrate, and examined by transmission electron microscopy (Tecnai G2 Spirit Bio-TWIN).

### 2.8. Detection of MDA Levels

ARPE-19 cells were collected, and the levels of MDA were tested by the MDA assay kit according to the manufacturer's instruction (Beyotime, Shanghai, China). The absorbance of standard and test products was detected at a wavelength of 530 nm.

### 2.9. Detection of SOD and GSH

The activity of SOD and the level of GSH were measured by the assay kits according to the manufacturer's instruction (Beyotime, Shanghai, China). The intracellular SOD activity was detected at a wavelength of 450 nm, and the intracellular GSH level was detected at a wavelength of 410 nm.

### 2.10. Enzyme-Linked Immunosorbent Assay (ELISA)

TNF-*α* and IL-1*β* levels were measured by the double antibody sandwich ELISA methods. Purified Human TNF-*α* and IL-1*β* antibodies were coated into the microtiter plate wells in advance. The samples were added into the wells and then combined with the antibody with HRP labeled. After the wells were washed completely, the TMB substrate solution was added. TMB substrate would become blue in the presence of HRP enzyme, and the reaction could be terminated by the sulphuric acid solution. The color variation could be measured spectrophotometrically at a wavelength of 450 nm. The concentrations of TNF-*α* and IL-1*β* in the samples were determined by comparing the optical density (OD) values of the samples with the standard curve.

### 2.11. Caspase 3 Activity Detection

Intracellular caspase 3 activity was detected by the caspase 3 activity assay kit. Briefly, APRE-19 cells were pretreated with NaHS for 30 min and subsequently treated with H_2_O_2_ for 24 h, and then, cells were collected by centrifugation at 600 g at 4°C for 5 min, washed with PBS, and then lysed in ice bath for 15 minutes. After centrifugation at 20000 g at 4°C for 15 min, the supernatant was incubated with Ac-DEVD-*ρ*NA for 1 h. The absorbance of the samples was detected at a wavelength of 405 nm.

### 2.12. Measurement of Autophagy Levels and Autophagy Flux

Autophagy levels and autophagy flux were measured using mCherry-EGFP-LC3 probes in ARPE-19 cells. ARPE-19 cells were transfected with mCherry-EGFP-LC3 adenoviruses at a multiplicity of infection (MOI) of 20. One day later, ARPE-19 cells were pretreated with NaHS for 30 min and then incubated with H_2_O_2_ for 1 h. Then, the fluorescent signals were detected by a confocal microscope (Zeiss 880 LSM 880).

### 2.13. Live Cell Imaging

ARPE-19 cells were cultured in 6-well plates, treated with the corresponding drugs, and observed with an inverted fluorescent microscope (Olympus IX71).

### 2.14. Hoechst 33342 and PI Stain

ARPE-19 cells were seeded in 6-well plates, treated with the corresponding drugs, and incubated with Hoechst 33342/PI in the dark for 10 min, before being observed under an inverted fluorescent microscope (Olympus IX71).

### 2.15. Statistical Analysis

Statistical analysis was performed as the mean ± standard deviation (SD). At least three independent experiments were conducted. Data analysis was expressed using Prism 8.0 software (GraphPad Software) and Microsoft Excel 2019. Data were analyzed using Student's *t*-test. Differences with *P* < 0.05 were considered statistically significant.

## 3. Results

### 3.1. Exogenous Hydrogen Sulfide Protects ARPE-19 Cells from H_2_O_2_-Induced Oxidative Damage

**Figure 1:**
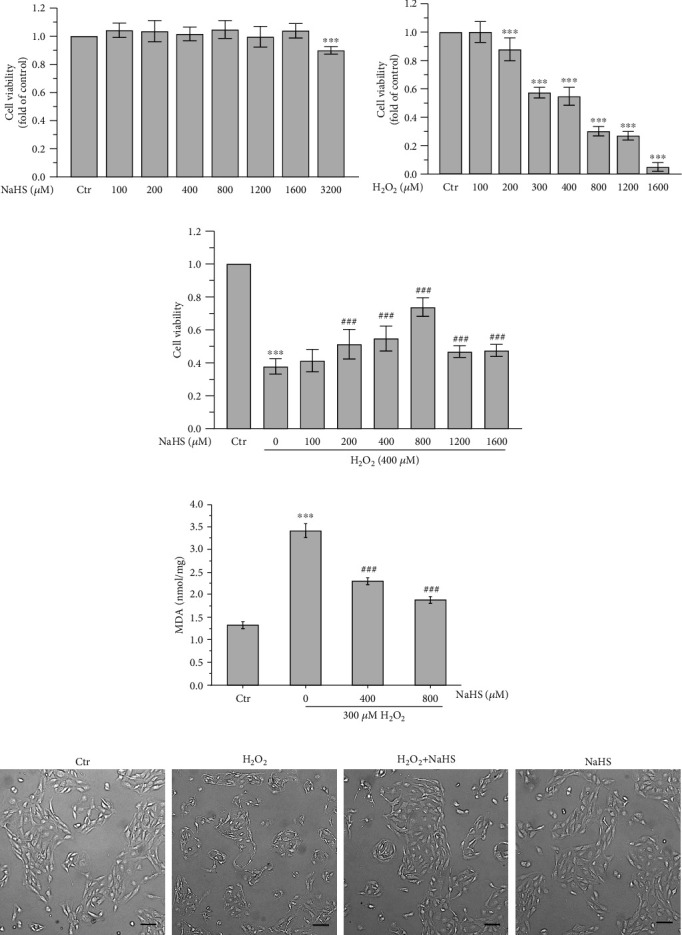
H_2_S protects ARPE-19 cells from H_2_O_2_-induced oxidative damage. (a) MTT assay was performed to detect the cytotoxicity of different concentrations of NaHS in ARPE-19 cells. (b) MTT assay was performed to measure the cytotoxicity of different concentrations of H_2_O_2_ in ARPE-19 cells. (c) ARPE-19 cells were treated with different concentrations of NaHS and 400 *μ*M H_2_O_2_. MTT assay was performed to examine the viability of ARPE-19 cells after H_2_O_2_ exposure for 24 h. (d) Lipid peroxide degradation product MDA was measured using TBA assay after being pretreated with NaHS for 30 min and then exposed to H_2_O_2_ for 24 h. (e) Cell morphology was examined in a bright field under an inverted fluorescent microscope after being pretreated with NaHS for 30 min and then exposed to H_2_O_2_ for 24 h. Scale bar = 100 *μ*m. Values are the mean ± SD. ^∗∗∗^*p* < 0.001 versus to the control group; ^###^*p* < 0.001 versus the H_2_O_2_ treatment alone group.

To examine the cytotoxic effect of hydrogen sulfide and H_2_O_2_ in cultured RPE cells, the cells were exposed to various concentrations of NaHS (100, 200, 400, 800, 1200, 1600, and 3200 *μ*M) for 24 h or H_2_O_2_ (100, 200, 300, 400, 800, and 1600 *μ*M) for 24 h. NaHS with 0-1600 *μ*M concentrations exhibited no obvious cytotoxicity to ARPE-19 cells (Figure 1(a)). ARPE-19 cell viability presented a dose-dependent manner with exposure to H_2_O_2_ and was of approximately 50% loss when cells were exposed to 300~400 *μ*M H_2_O_2_ (Figure 1(b)). Thus, H_2_O_2_ with 300~400 *μ*M concentration was selected for the subsequent experiments. It has been observed that 800 *μ*M NaHS significantly attenuated the reduction in ARPE-19 viability caused by H_2_O_2_ (Figure 1(c)). Moreover, the protective effects of H_2_S on H_2_O_2_-induced oxidative damage were further evaluated by the level of MDA. The content of MDA in cells is often used as an index to evaluate the degree of oxidative damage in cells [16]. The results showed that H_2_O_2_ treatment induced the increase of MDA, which was dramatically inhibited by the NaHS pretreatment (Figure 1(d)), demonstrating the protective effect of H_2_S on oxidative damage. Then, the cell morphology was examined. As shown in Figure 1(e), H_2_S significantly attenuated the morphological damage of cells induced by H_2_O_2_. These results demonstrate that exogenous H_2_S protected ARPE-19 cells against H_2_O_2_-induced oxidative injury.

### 3.2. Exogenous Hydrogen Sulfide Inhibits H_2_O_2_-Induced Oxidative Stress and Inflammation in ARPE-19 Cells

**Figure 2:**
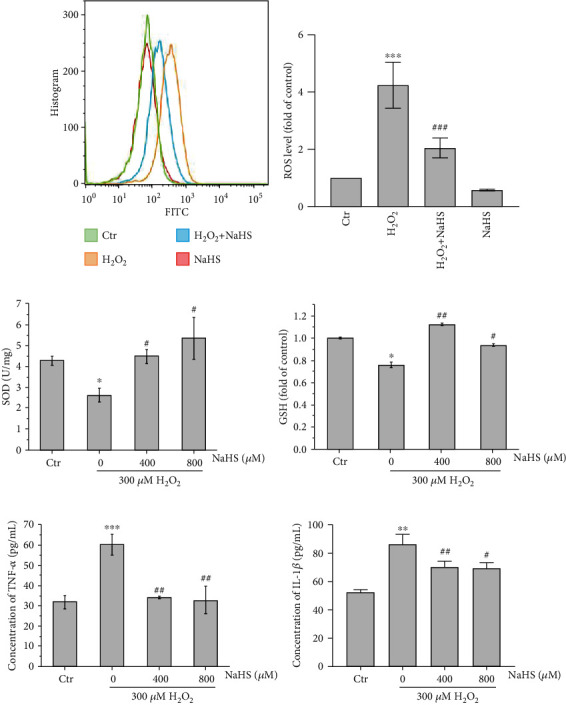
H_2_S protects ARPE-19 cells against H_2_O_2_-induced oxidative stress. (a) ARPE-19 cells were pretreated with NaHS for 30 min and exposed to H_2_O_2_ for 1 h. ROS level was detected using DCFH-DA by flow cytometry. (b) Statistics on the ROS level. (c) Intracellular SOD activity was detected by the assay kit. (d) Intracellular GSH level was measured by the assay kit. (e, f) ELISA detection of secretion of inflammatory factors TNF-*α* and IL-1*β*. Cells were pretreated with NaHS for 30 min and then exposed to H_2_O_2_ for 24 h. Values are the mean ± SD. ^∗^*p* < 0.05 and ^∗∗∗^*p* < 0.001 versus the control group, ^#^*p* < 0.05, ^##^*p* < 0.01, and ^###^*p* < 0.001 versus the H_2_O_2_ treatment alone group.

Intracellular ROS and inflammation played a vital role in diverse types of cells, closely related to AMD [17, 18]. We evaluated ROS levels via flow cytometry and inflammation cytokines (TNF-*α*, IL-1*β*) via ELISA to explore the effects of H_2_S on ROS generation and inflammation. It was shown that H_2_O_2_ increased the ROS level in ARPE-19 cells and H_2_S exhibited a significant inhibitory effect on H_2_O_2_-induced ROS production (Figures 2(a) and 2(b)). Furthermore, the impacts of H_2_S on the antioxidant enzyme (SOD) activity and the intracellular antioxidant molecule (GSH) level in ARPE-19 cells were also investigated. The data revealed that H_2_S attenuated the reduction of intracellular SOD activity and GSH level caused by H_2_O_2_ (Figures 2(c) and 2(d)). In addition, H_2_S significantly reduced the secretion increase of cytokines induced by H_2_O_2_ (Figures 2(e) and 2(f)). Thus, H_2_S could suppress ROS generation and inflammatory cytokine secretion, and it also increased SOD and GSH levels, which might account for its protective effects.

### 3.3. Exogenous Hydrogen Sulfide Protects ARPE-19 Cells against H_2_O_2_-Induced Apoptosis

**Figure 3:**
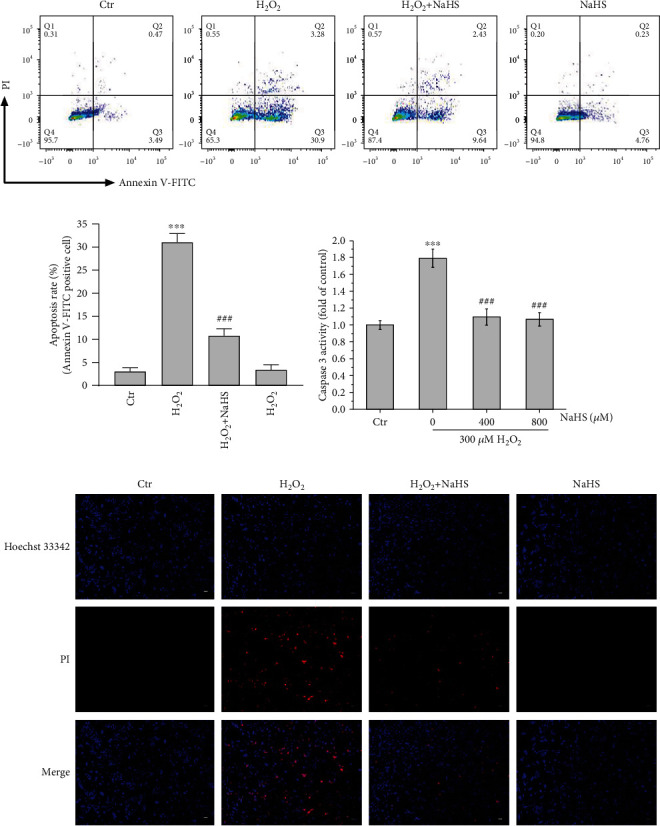
H_2_S protects ARPE-19 cells from H_2_O_2_-induced apoptosis. (a, b) Cell apoptosis was analyzed with Annexin V-FITC and PI stain. (c) Intracellular caspase 3 activity was measured by the caspase 3 kit. (d) Cells were stained with PI and Hoechst 33342. Scale bar = 100 *μ*m. Values are the mean ± SD. ^∗∗∗^*p* < 0.001 versus the control group; ^###^*p* < 0.001 versus the H_2_O_2_ treatment alone group.

To further investigate whether H_2_S protects against H_2_O_2_-induced cell death through an antiapoptotic effect, cell apoptosis was evaluated by flow cytometry using Annexin V-FITC/PI. The results showed that the proportion of Annexin V-FITC and PI-positive cells exhibited a statistically significant increase in the ARPE-19 cells treated with H_2_O_2_ for 24 h alone and that NaHS pretreatment significantly reduced the proportion of cells apoptosis (Figures 3(a) and 3(b)). Moreover, H_2_S significantly attenuated the increase of caspase 3 activity induced by H_2_O_2_ (Figure 3(c)). Cell morphology was also investigated. Hoechst 33342 stains the nucleus of ARPE-19 cells with blue fluorescence, and PI stains death cells with red fluorescence; therefore, the red fluorescence represents cell death. NaHS pretreatment reduced PI-positive cells, demonstrating cell death was inhibited by NaHS pretreatment (Figure 3(d)). Taken together, these results indicated that H_2_S protected ARPE-19 cells against H_2_O_2_-induced cell death/apoptosis.

### 3.4. Exogenous Hydrogen Sulfide Decreases H_2_O_2_-Induced Autophagy in ARPE-19 Cells

**Figure 4:**
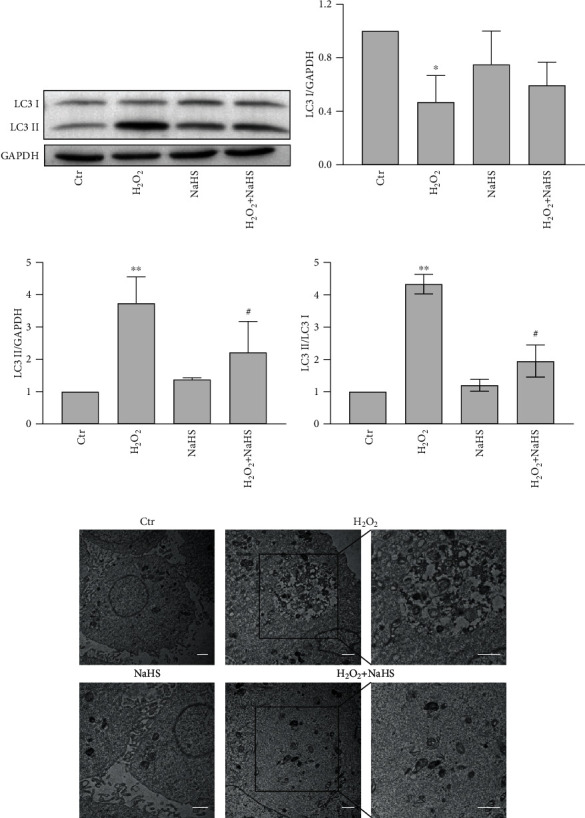
H_2_S decreases H_2_O_2_-induced occurrence of autophagy in ARPE-19 cells. (a) ARPE-19 cells were pretreated with NaHS for 30 min and then treated with H_2_O_2_ for 1 h. The protein expression and transform of LC3 I and LC3 II in ARPE-19 cells were analyzed by Western blot. The quantitative analyses of LC3 I/GAPDH, LC3 II/GAPDH, and LC3 II/LC3 I are shown (b–d). (e) Detection of intracellular autophagic vesicles by TEM after being pretreated with NaHS for 30 min and then treated with H_2_O_2_ for 24 h. Scale bar = 1 *μ*m. Values are the mean ± SD. ^∗∗^*p* < 0.01 versus the control group; ^#^*p* < 0.05 versus the H_2_O_2_ treatment alone group.

**Figure 5:**
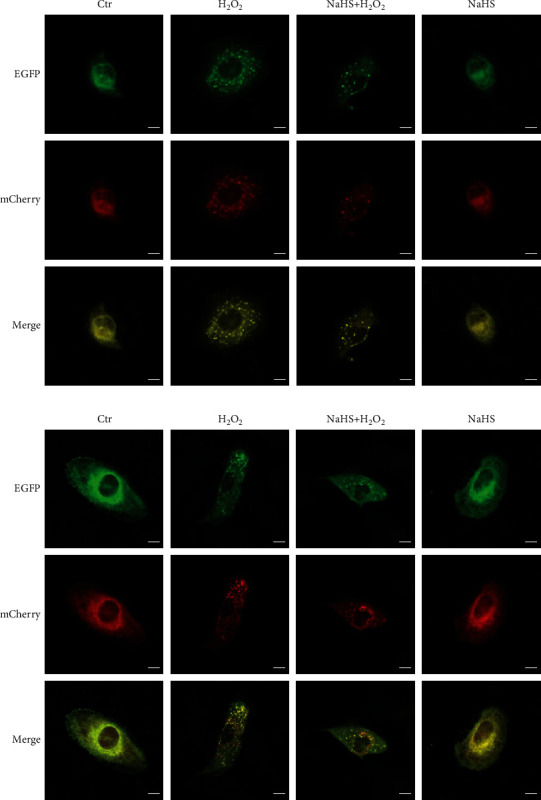
H_2_S decreases H_2_O_2_-induced autophagic flux in ARPE-19 cells. (a) ARPE-19 cells were pretreated with NaHS for 30 min and then treated with H_2_O_2_ for 1 h. The fluorescent mCherry-EGFP-LC3B signal in the cell was used to detect autophagosomes by the confocal microscope. (b) Cells were pretreated with NaHS for 30 min and then treated with H_2_O_2_ for 24 h. Scale bar = 10 *μ*m.

It has been reported that oxidative stress can induce autophagy, which is also closely related to apoptosis [19]. Thus, the protection of ARPE-19 cells against oxidative stress may involve autophagy. Therefore, the impacts of H_2_O_2_ and H_2_S on the level of autophagy in ARPE-19 cells were investigated. LC3B distribution and processing is a classical autophagic marker, and the ratio of conversion from LC3 I to LC3 II is closely correlative with the extent of autophagosome formation. Western blot analysis revealed that H_2_O_2_ significantly induced the conversion of LC3 I to LC3 II, which was significantly reduced by NaHS pretreatment (Figures 4(a)–4(d)). Transmission electron microscope studies showed that H_2_O_2_ treatment increased the number of intracellular autophagic vesicles and that NaHS pretreatment reduced the autophagic vesicles (Figure 4(e)). Additionally, the autophagy formation was monitored using mCherry-EGFP-LC3 adenoviruses. It was shown that H_2_O_2_ significantly increased the number of autophagosomes (yellow puncta) and that NaHS pretreatment effectively decreased the autophagosome number (Figure 5). No fusion of autophagosomes and lysosomes was seen at the early stage (Figure 5(a)). But when cells were treated with H_2_O_2_ for 24 h, the fusion of autophagosomes and lysosomes in cells were observed (red puncta), which was significantly reduced by NaHS pretreatment (Figure 5(b)). These results suggest that exogenous H_2_S decreased oxidative stress-induced autophagy in ARPE-19 cells.

**Figure 6:**
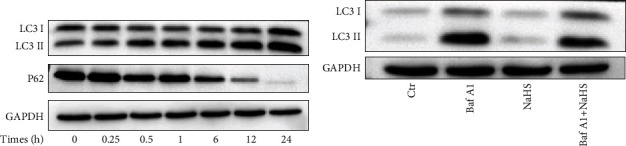
Further evidences show that H_2_O_2_ triggers autophagic flux and that H_2_S does not increase autophagic flux in ARPE-19 cells. (a) ARPE-19 cells were treated with 400 *μ*M H_2_O_2_ for gradient time (0~24 h), and then, cells were collected for Western blot analysis of the autophagy marker proteins LC3 I/II and P62. (b) ARPE-19 cells pretreated with 20 nM Baf A1 (inhibiting the fusion of autophagosomes and lysosomes) for 1 h and then treated with NaHS for 24 h. The LC3 I/II protein expression was also analyzed by Western blot.

However, there is a possibility that the accumulation of autophagic vesicles is due to H_2_O_2_-blocked autophagic flux. To exclude this possibility, the changes of autophagy binding protein P62 and LC3 II were monitored at the same time. P62 binds autophagosome membrane protein LC3/Atg8, aggregating the formation of autophagosome, and then is degraded along with the fusion of autophagosomes and lysosomes [20–22]. After H_2_O_2_ treatment, with the LC3 conversion from I-type into II-type, P62 was decreased gradually with increased time (0~24 h), illustrating that H_2_O_2_ increased the autophagic flux (Figure 6(a)).

There is another possibility that NaHS increases autophagic flux, causing a reduction in autophagic vesicles at the 24 h time point. To eliminate this possibility, the inhibitor Baf A1 was used to inhibit the fusion of autophagic vesicles and lysosomes. The autophagic flux was inhibited by Baf A1, leading to the accumulation of LC3 II [23, 24]. But NaHS did not aggravate this accumulation with cotreatment of Baf A1, illustrating NaHS did not increase the occurrence of autophagy (Figure 6(b)). Taken together, all the above results demonstrated that NaHS inhibited H_2_O_2_-triggered autophagic flux.

### 3.5. Autophagy Is Involved in H_2_O_2_-Induced Oxidative Stress and Cell Apoptosis

**Figure 7:**
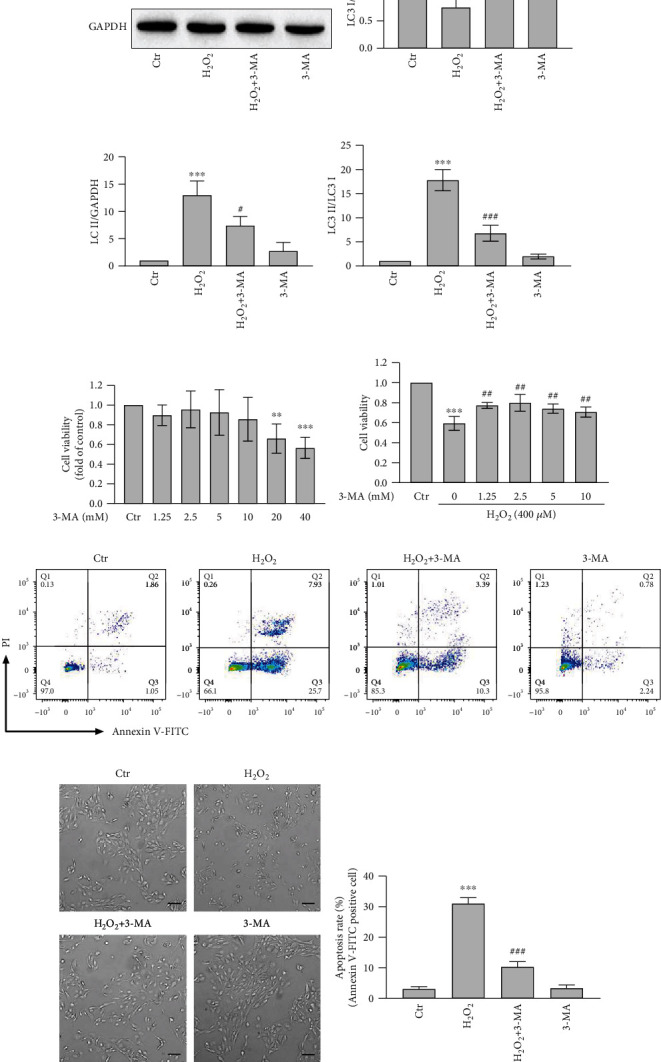
Autophagy is involved in H_2_O_2_-induced oxidative stress and cell apoptosis. (a) ARPE-19 cells were pretreated with 3-MA for 3 h and then treated with H_2_O_2_ for 1 h. The protein expression and transform of LC3 I and LC3 II in ARPE-19 cells were analyzed by Western blot. (b–d) The quantitative analyses of LC3 I/GAPDH, LC3 II/GAPDH, and LC3 II/LC3 I are shown. (e) MTT assay was performed to detect the cytotoxicity of different concentrations of the autophagy inhibitor 3-MA for 24 h in ARPE-19 cells. (f) ARPE-19 cells were treated with different concentrations of the autophagy inhibitor 3-MA and 400 *μ*M H_2_O_2_. MTT assay was performed to examine the viability of ARPE-19 cells after ARPE-19 cells were pretreated with 3-MA for 3 h and then exposed to H_2_O_2_ for 24 h. (g, i) Cell apoptosis was analyzed with Annexin V-FITC and PI stain by flow cytometry. (h) Cell morphology was examined in a bright field under an inverted fluorescent microscope after ARPE-19 cells were pretreated with 3-MA for 3 h and then exposed to H_2_O_2_ for 24 h. Scale bar = 100 *μ*m. Values are the mean ± SD. ^∗∗^*p* < 0.01 and ^∗∗∗^*p* < 0.001 versus the control group; ^###^*p* < 0.001 versus the H_2_O_2_ treatment alone group.

**Figure 8:**
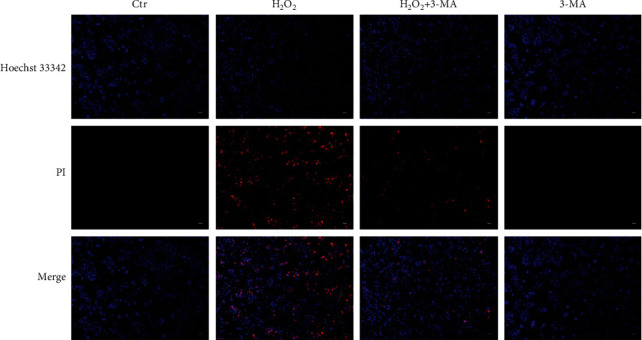
3-MA inhibits H_2_O_2_-induced cell death by PI/Hoechst 33342 staining. The autophagy inhibitor 3-MA ameliorates cell morphological damage induced by H_2_O_2_. ARPE-19 cells were stained with PI and Hoechst 33342 after being pretreated with 3-MA for 3 h and then exposed to H_2_O_2_ for 24 h. Scale bar = 100 *μ*m.

To investigate whether autophagy is related to oxidative damage, another autophagy inhibitor 3-MA was used to regulate autophagy in ARPE-19 cells. 3-MA inhibits autophagy upstream signal PI3K, leading to the inhibition of the conversion of LC3 I to LC3 II and autophagic vesicle formation [25]. The present study doubtlessly showed 3-MA inhibited the conversion of LC3 I to LC3 II (Figures 7(a)–7(d)). Moreover, after autophagy was inhibited by 3-MA, the decrease of cell viability mediated by H_2_O_2_ was obviously attenuated (Figures 7(e) and 7(f)). It was also shown that 3-MA could improve cell morphology damage by H_2_O_2_ (Figure 7(h)). Furthermore, the inhibition of autophagy by 3-MA inhibited cell apoptosis mediated by H_2_O_2_ (Figures 7(g) and 7(i)). And Hoechst 33342/PI staining also showed that the inhibition of autophagy by 3-MA improved cell survival (Figure 8). In summary, these results indicated that the inhibition of autophagy by 3-MA reduced the oxidative damage and apoptosis induced by H_2_O_2_.

## 4. Discussion

Although extensive research has shown that oxidative stress and cell apoptosis of RPE cells may play a crucial role in the pathogenesis of AMD, the mechanisms of oxidative stress-induced RPE cell death and the exact relationship between oxidative damage and AMD remain elusive [26–28]. It is a research hotspot for studying how to design approaches to protect RPE cells from oxidative stress and apoptosis as therapeutic options for slowing down AMD. H_2_S is well-recognized as a second messenger. Accumulated evidence reveals that H_2_S provides enzymatic antioxidant function [29–31]. But it is currently poorly understood whether H_2_S can protect RPE cells from oxidative damage. In the present study, we observed that the viability of ARPE-19 cells was inhibited when exposed to H_2_O_2_, but H_2_S pretreatment significantly attenuated H_2_O_2_-induced oxidative damage (Figures 1(c) and 1(e)). Interestingly, 1200~1600 *μΜ* H_2_S is less effective in protecting against the reduction of cell viability, compared to 800 *μΜ* H_2_S (Figure 1(c)), which was probably due to that high concentrations of H_2_S causing side effects on cells, although we did not detect obvious cell viability changes (Figure 1(a)). More and more studies show that ROS and inflammation have essential roles in the progress and development of early AMD and underlie many diseases including AMD [2, 32, 33]. However, the effects of H_2_S on ROS and inflammation involved in ARPE-19 cells and the pathogenesis of AMD are unknown [2, 3, 32, 33]. This study indicates that the exposure of ARPE-19 cells to H_2_O_2_ results in ROS generation and inflammatory cytokine secretion, but these effects are significantly ameliorated by NaHS pretreatment (Figures 2(a), 2(e) and 2(f)).

Previous research has reported that H_2_S has tremendous potential in the treatment of a wide range of physiological and pathological processes including age-related diseases [34]. H_2_S is endogenously generated by several enzymes in mammals, including cystathionine *β*-synthase (CBS), cystathionine *γ*-lyase (CSE), and 3-mercaptopyruvate sulfurtransferase (3MST) [34–36]. H_2_S level and expression of its endogenous enzymes CBS, CSE, and 3MST in retinal tissues are significantly decreased along with the loss of retinal ganglion cells (RGCs) in a chronic ocular hypertension rat model [34–36]. Exogenous H_2_S influenced the expression of antioxidant enzymes CSE and SOD to protect against oxidative stress and myocardial fibrosis [37]. H_2_S also improved enzymatic antioxidant function by mediating the activities of Gpx, SOD, and CAT [30]. This study has shown that H_2_S improves the SOD activity and GSH level inhibited by H_2_O_2_ in ARPE-19 cells (Figures 2(c) and 2(d)). H_2_O_2_-induced apoptosis of RPE cells is a common model for oxidative stress [38–40]. In the present study, H_2_O_2_ increased the activity of apoptosis-related protein caspase 3 in ARPE-19 cells and significantly increased the rate of apoptosis. Instead, H_2_S pretreatment significantly inhibited the apoptosis rate and reduced the activity of caspase 3 (Figures 3(a) and 3(c)).

Autophagy, as a catabolic process, is considered to protect the cells against various factors of stress and is aimed at recycling cytoplasmic components and damaged organelles caused by diverse stress [41, 42]. A lot of evidence reveals oxidative stress-mediated occurrence of autophagy in diverse kinds of cells [43], including ARPE-19 cells. It is reported that autophagy plays a positive role in promoting cell survival and anti-apoptosis [44–49]. However, in the present study, autophagy plays a negative role to enhance H_2_O_2_-induced RPE cell damage and apoptosis. The autophagy inhibitor 3-MA suppresses the early formation of autophagy and significantly attenuates cell viability inhibition and cell apoptosis induced by H_2_O_2_ (Figures 7(e)–7(i)). According to previous evidence, not only the inhibition of autophagy protects cells from oxidative damage but also the stimulation of autophagy increases apoptosis [50–56]. Therefore, we speculate that the oxidative stress caused by H_2_O_2_ might trigger high-level oxidative damage through inducing an excessively high level of autophagy, but more evidence is needed.

Next, we wanted to confirm whether the effect of H_2_S against oxidative stress involved autophagy in ARPE-19 cells. Some studies showed that high concentration H_2_S promoted autophagy, but some researches revealed that H_2_S attenuated the process of autophagy [13, 57]. Furthermore, multiple signaling pathways were involved in the process of autophagy in H_2_S-treated cells [13, 57, 58]. In the present study, Western blot results suggested that H_2_S pretreatment reduced the conversion of LC3 I to LC3 II and transmission electron microscopy also confirmed the same conclusion that H_2_S could inhibit autophagy. The detection of autophagy flux further proved that H_2_S could reduce the level of autophagy (Figures 4(a)–4(e), 5(a) and 5(b)). Meanwhile, P62 was decreased gradually with the LC3 conversion from I-type into II-type, illustrating that H_2_O_2_ increased the autophagic flux (Figure 6(a)). And NaHS did not aggravate the LC3 II accumulation with cotreatment of Baf A1, showing NaHS did not increase the occurrence of autophagy (Figure 6(b)). These results further confirmed that NaHS inhibited H_2_O_2_-triggered autophagic flux.

However, it is not clear whether H_2_S directly or indirectly affects the regulation of autophagy level, which is also the main direction of our next research. Previous researches reported that the intervention of some drugs changed the intracellular ROS level and thus altered the autophagy level affected by ROS [47, 59]. Consequently, determining the regulatory role of H_2_S on autophagy might be crucial to delay the occurrence of AMD.

In the present study, low confluent ARPE-19 cells (1 × 10^5^ cells/well) are selected to guarantee their sufficient nutrition, in accordance with previous reports [3, 23, 60–63]. But it should be emphasized that in real life, the RPE is a confluent monolayer and would probably react very differently to the above-mentioned stressors. Therefore, determining the protective effect of H_2_S on confluent ARPE-19 cells should be in our future studies.

## 5. Conclusion

All in all, exogenous H_2_S has protective effects against H_2_O_2_-induced intracellular ROS generation, oxidative damage, inflammatory factors secretion, antioxidant level decrease, cell morphological alteration, cell survival inhibition, and apoptosis in retinal ARPE-19 cells. Moreover, H_2_O_2_ triggers the intracellular autophagy level, which is inhibited by H_2_S pretreatment. The autophagy inhibitor also suppresses H_2_O_2_-induced oxidative damage and apoptosis. Therefore, our results reveal that autophagy is involved in the protection of H_2_S against oxidative stress-triggered apoptosis in retinal ARPE-19 cells. These findings suggest that exogenous H_2_S has a potential value in the treatment of AMD.

## Data Availability

The data used to support the findings of this study are available from the corresponding author upon request.
